# Ethanol is indispensable for virucidal hand antisepsis and without toxic risks in daily use

**DOI:** 10.3205/dgkh000428

**Published:** 2023-01-23

**Authors:** Axel Kramer, Hicham Benkhai, Christian Jäkel, Paula Zwicker

**Affiliations:** 1 Institute of Hygiene and Environmental Medicine, University Medicine Greifswald, Germany; 2 Azyro, Luxembourg; 3 Kanzlei Dr. Jaekel, Medical law – Pharmaceuticals law – Medical devices law, Lübben (Spreewald), Germany

**Keywords:** biocidal product regulation, 1-propanol, 2-propanol, ethanol, ethanol based hand rubs, benefit-risk-assessment, virucidal efficacy, dermal absorption, worker safety, patient safety

## Abstract

The approval of ethanol by the Biocidal Products Regulation has been under evaluation since 2007 due to controversial opinions on the risk assessment. Because of this critical situation, 2022 a memorandum was published to verify whether the use of ethanol for hand antisepsis poses any hazard. On the basis of the memorandum a toxicological evaluation of ethanol-based hand rubs is given.

## Abstract (de)

Die Bewertung von Ethanol als Biozid gemäß der Biozid-Verordnung wird seit 2007 aufgrund kontroverser Meinungen zur Risikobewertung geprüft. Wegen dieser kritischen Situation wurde 2022 ein Memorandum veröffentlicht, um zu überprüfen, ob die Verwendung von Ethanol zur Händedesinfektion eine Gefahr darstellt. Auf der Grundlage des Memorandums wird eine zusammenfassende toxikologische Bewertung Ethanol basierten Händedesinfektionsmittel gegeben.

## Introduction

Biocidal products legislation in Europe aims to reduce the number and quantity of chemicals used. However, ensuring hygiene in healthcare facilities is not an objective of European biocidal products legislation. This results in an unresolvable conflict of objectives between reducing the number and quantity of chemicals and the need to use disinfectants in sufficient variety and quantity to prevent infections in healthcare facilities. 

Because of this critical situation, on base of a comprehensive literature search to verify whether the use of ethanol for hand antisepsis poses a hazard of reproductive toxicity, a memorandum was published [1]. At the same time, the memorandum stated that among the active ingredients used in hand antiseptics, only ethanol is effective against non-enveloped viruses, but not propanol and isopropanol [1].

## Legal and regulatory background

Alcohol-based hand rubs (ABHR) are classified as biocidal products in the European Union and the European Economic Area. For 2-propanol, this classification was explicitly made by the “Implementing Decision (EU) 2016/904 according to Article 3(3) of Regulation (EU) No 528/2012 on propan-2-ol containing products used for hand disinfection” [2]. This classification applies to both hygienic hand rub and surgical hand preparation. Hand rubs are classified as product type (PT) 1 according to Annex V of Regulation (EU) No 528/2012 (Biocidal Products Regulation) [3]. Products used for the disinfection of surfaces are also biocidal products. Therefore, they are classified as PT 2 according to Annex V of the Biocidal Products Regulation [3]. Merely disinfectants intended for the disinfection of medical devices are classified as medical devices according to Regulation (EU) No. 2017/745 (Medical Device Regulation) [4]. 

According to the requirements of the Biocidal Products Regulation, active substances for biocidal products must be approved. With Delegated Regulation (EU) No. 1062/2014 [5], the European Commission has defined a list of active substances that are being evaluated concerning their possible approval for use in biocidal products.

Approval is granted in each case depending on the scope of application for the product types in accordance with Annex V of the Biocidal Products Regulation 

Based on the approval as an active substance for use in biocidal products, all biocidal products – except for biocidal products that can be marketed under transitional provisions – require authorization for their marketability. In principle, there is the national authorization, the mutual recognition procedure for national authorizations (when a product is marketed in several Member States) and the Union authorization. For certain products that do not contain substances of concern, for example, there is a simplified procedure. There is also the option to apply for authorization of a biocidal product that is either identical to an already authorized biocidal product or similar to a biocidal product for which an application for authorization is ongoing. To evaluate efficacy in authorization procedures, ECHA has issued guidelines containing general requirements and requirements for individual product types. These guidelines also include requirements for the efficacy of hand rubs.

## The current classification of alcohol-based hand rubs

2-Propanol was approved by means of Implementing Regulation (EU) 2015/407 [6] as an active substance for use in biocidal products of PT 1 (human hygiene), PT 2 (disinfectants and algaecides not intended for direct application to humans or animals) and PT 4 (food and feed area). Thirteen PT 1 biocidal products, 70 PT 2 biocidal products and 38 PT 4 biocidal products are approved in the EU with the active ingredient 2-propanol. For 2-propanol, the evaluation lasted from 2007 to 2015. 

1-Propanol was approved as an active substance for use in biocidal products of PT 1, 2 and 4 by means of Implementing Regulation (EU) 2017/2001 [7]. In the EU, three biocidal products of PT 1, four biocidal products of PT 2 and four biocidal products of PT 4 are approved with the active substance 1-propanol (in each case in combination with 2-propanol). For 1-propanol, the evaluation lasted from 2007 to 2017. 

The classification of ethanol has been paradoxically since 2007 not finalized due to the contradictory perceptions of experts and authorities. As a result, the evaluation for the active substance ethanol is far behind schedule. The rapporteur Member State for the evaluation of ethanol is Greece. Ethanol is a candidate for active substance substitution as defined in Article 10 of Regulation (EU) No 528/2012 in PT 1, 2 and 4 [3]. 

Where appropriate, ethanol should be classified as a carcinogen within the meaning of Regulation (EC) No 1272/2008 (CLP Regulation) (8). According to ECHA, there is no general agreement among data submitters, but a minority indicates that they consider this substance as carcinogenic (14.28% of REACH registrations). Of the minority indicating the property of concern, most indicate that it may relate to an impurity or additive rather than the substance itself. 

In the “Registry of intention” on classification and labeling, the Greek authority updated a harmonized classification and labeling of ethanol on 27 July 2020 [3]. The current intention of extending harmonized classification provides, among other things, a classification as reproductive toxicity category 2 (“suspected to have CMR potential for humans”; carcinogen, mutagen, reprotoxic-CMR). This is a downgrade from the classification as carcinogenic category 1A and reproductive toxicity 1A. However, it is essential to note that the Risk Assessment Committee of the ECHA is not bound by the proposed classification. Hence the classification of ethanol as carcinogenic and/or reproductive toxicity category 1 by the ECHA still cannot be excluded. The consequence of classifying ethanol as CMR would have been that the only active ingredient in hand antiseptics with efficacy against non-enveloped viruses would no longer be available. 

Considering the current open assessment of ethanol as a biocide – since 2007 the approval of ethanol as a biocide is under evaluation –, in July 2022, the journal *Antimicrobial Resistance and Infection Control* published a memorandum from the alcohol-based hand rub Task Force, WHO Collaborating Centre on Patient Safety, and the Commission for Hospital Hygiene and Infection Prevention at the Robert Koch Institute, Berlin, Germany [1].

## Synopsis of the toxicological evaluation of ethanol-based hand rubs

The evidence for a reprotoxic effect of ethanol originates from pregnant women’s consumption of alcoholic beverages with incomparably higher ethanol uptake [8]. However, there is no epidemiological hint of toxicity for workers from handling ethanol-containing products in industry or using ethanol-based hand rubs in healthcare facilities [9]. 

Excessive hand antisepsis using an ethanol-based hand rub led to an blood level of less than 50 mg/L [8]. Another study revealed between 0.5% and 2.3% ethanol uptake after hygienic or surgical hand antisepsis resulting in a maximum blood level of 30.10 mg/L [10]. In contrast, beer consumption (0.5 g per kg body weight) can lead to a blood ethanol level of up to 503±98 mg/L [11]. In comparison: fruit juices are allowed to contain up to 3 g/L ethanol [12]; indeed, between 1.1 and 3.9 g/L were found [13]. Furthermore, alcohol-free beer, allowed to contain 0.5% vol was found to contain up to 4.94% vol. Assuming a resorption rate of 90%, Bonte et al. calculated a blood concentration of 0.17‰ when drinking 0.5 l juice for a man weighting 75 kg [14]. However, this is a sum parameter not comparable to a peak blood alcohol level as given before.

The Poisindex^®^ [15] classifies ethanol as a carcinogenicity category: A3. This means the agent is carcinogenic in experimental animals at a relatively high dose, by route(s) of administration, at site(s), of histologic type(s), or by mechanism(s) that may not be relevant to worker exposure. Available epidemiologic studies do not confirm an increased cancer risk in exposed humans. Available evidence does not suggest that the agent is likely to cause cancer in humans except under uncommon or unlikely routes or levels of exposure [15].

In conclusion of animal studies, epidemiologic analysis and the risk assessment of the absorbed ethanol by medically indicated hand rub, there is no risk of carcinogenicity, mutagenicity or reprotoxicity from repeated use of ethanol-based hand rubs as a hand antiseptic [16].

## Indispensable importance of ethanol for hand antisepsis

Non-enveloped viruses are unequally more stable against chemical agents than enveloped viruses. Of the three alcohols used in ABHR (ethanol, 2-propanol and 1-propanol), only ethanol-based formulations proved to be effective within 30–60 s against non-enveloped viruses such as adeno-, polio-, human entero-, human papilloma-, polyoma-, echo- and coxsackie viruses [1]. Since non-enveloped viruses have been detected on hands with a tenacity of up to 7 h [17] and induced nosocomial infections as well as nosocomial and foodborne outbreaks, i.e. norovirus outbreaks on cruise ships, virucidal hand antisepsis is indispensable to control nosocomial infections and outbreaks by non-enveloped viruses. Should a non-enveloped virus, instead of enveloped influenza viruses or SARS-CoV-2, cause a pandemic, when not using ethanol, there would be no sufficiently effective ABHR to prevent transmission.

## Conclusions

The memorandum insistently recommends maintaining ethanol as a biocidal active ingredient in hand antiseptics for use in healthcare settings as well as in food industry and in public areas to prevent infections by non-enveloped viruses. There are no alternatives to challenge the use of ethanol-based hand rubs [1].

## Notes

### Competing interests

The authors declare that they have no competing interests.

### Authors’ ORCID

- Axel Kramer: 0000-0003-4193-2149
- Paula Zwicker: 0000-0001-8891-7160
